# Supplementary material for: "This is an absolute witch-hunt for nursing, and nobody feels safe." A qualitative study of nurses, mental health, and the criminal prosecution of a nurse error
Source: PLOS Ment Health. 2024 Dec 12;1(7):e0000178. doi: 10.1371/journal.pmen.0000178 (PMC12798251; doi:10.1371/journal.pmen.0000178)
Supplement: Attachment — Submitted filename: Response to reviewers.docx [file pmen.0000178.s001.docx]

We would like to thank the reviewers for their thoughtful review and helpful suggestions. We have addressed the comments below in red, which are also tracked in the uploaded revised manuscript. A clean, untracked manuscript has also been submitted as requested. Thanks so much for considering our research on this important topic.

Journal Requirements:

1. In the ethics statement in the Methods, you have specified that verbal consent was obtained. Please provide additional details regarding how this consent was documented and witnessed, and state whether this was approved by the IRB Our IRB approved the use of a cover letter consent. In this study, the cover letter is either emailed to the participant or given to them as a hard copy before the interview is conducted. The cover letter contains all elements of informed consent but does not require a signature. The interviewer always confirms consent verbally with each participant before the interview begins and all questions have been answered. We added a sentence about the cover letter and the consent process is described in the methods section.

2. In the online submission form, you indicated that [The anonymized interviews are available from the corresponding author with reasonable request.].

All PLOS journals now require all data underlying the findings described in their manuscript to be freely available to other researchers, either 
a. In a public repository, 
b. Within the manuscript itself, or 
c. Uploaded as supplementary information.

This policy applies to all data except where public deposition would breach compliance with the protocol approved by your research ethics board. If your data cannot be made publicly available for ethical or legal reasons (e.g., public availability would compromise patient privacy), please explain your reasons by return email and your exemption request will be escalated to the editor for approval. Your exemption request will be handled independently and will not hold up the peer review process, but will need to be resolved should your manuscript be accepted for publication. One of the Editorial team will then be in touch if there are any issues. We feel that publicly uploading our interview transcripts may jeopardize the privacy of our participants. This is a controversial topic, and to encourage freedom of thoughts and ideas, our participants were assured their participation would be kept with the most confidence. Specific errors were disclosed that could lead back to their identity as participants, thus we are requesting an exemption to publish the transcripts or upload them to a public repository. However, we are willing to share them with individual researchers with reasonable requests and assurance of data protection and security. I will also send this request by email to the editorial board as requested.

3. Please review your reference list to ensure that it is complete and correct. If you have cited papers that have been retracted, please include the rationale for doing so in the manuscript text, or remove these references and replace them with relevant current references. Any changes to the reference list should be mentioned in the rebuttal letter that accompanies your revised manuscript. If you need to cite a retracted article, indicate the article’s retracted status in the References list and also include a citation and full reference for the retraction notice. We believe our references are complete.

Additional Editor Comments (if provided):

*Comments from PLOS Editorial Office:* In addition to the comments raised, the editorial office would like to request that you mention the caveat of the small sample size. Whilst we do appreciate that this is a qualitative paper, we feel it would be important to acknowledge that the experiences discussed may not reflect all experiences in the community . Thank you for the opportunity to respond to the comments below. We recognize the small sample size as one of our limitations. In addition, in response to the reviewers, we have added more descriptions of the limitations, including the potential of selection bias and the limitation of generalizability.

[Note: HTML markup is below. Please do not edit.]

Reviewers' comments:

Reviewer's Responses to Questions

**Comments to the Author**

1. Does this manuscript meet PLOS Mental Health’s [publication criteria](https://nam04.safelinks.protection.outlook.com/?url=https%3A%2F%2Fc05y1x9s.r.us-east-2.awstrack.me%2FL0%2Fhttps%3A%252F%252Fjournals.plos.org%252Fmentalhealth%252Fs%252Fcriteria-for-publication%2F1%2F010f01924ae67f7e-01a4755c-df8e-4cc1-bc38-b44f62b03448-000000%2FGeNqEzFH6i7cayZ6EYhVZsbTtiY%3D179&data=05%7C02%7Cpatricia.dekeseredy%40hsc.wvu.edu%7C035b795db0ae4b650fdc08dce283c400%7Ca2d1f95f851044248ae15c596bdbd578%7C0%7C0%7C638634302766988838%7CUnknown%7CTWFpbGZsb3d8eyJWIjoiMC4wLjAwMDAiLCJQIjoiV2luMzIiLCJBTiI6Ik1haWwiLCJXVCI6Mn0%3D%7C0%7C%7C%7C&sdata=2A5E1mOsKPOR4NXAmBWQD%2FqX2o3lTy5EMO%2Bwm0osjKM%3D&reserved=0)? Is the manuscript technically sound, and do the data support the conclusions? The manuscript must describe methodologically and ethically rigorous research with conclusions that are appropriately drawn based on the data presented.

Reviewer #1: Partly

Reviewer #2: Yes

2. Has the statistical analysis been performed appropriately and rigorously?

Reviewer #1: N/A

Reviewer #2: Yes

3. Have the authors made all data underlying the findings in their manuscript fully available (please refer to the Data Availability Statement at the start of the manuscript PDF file)?

The [PLOS Data policy](https://nam04.safelinks.protection.outlook.com/?url=https%3A%2F%2Fc05y1x9s.r.us-east-2.awstrack.me%2FL0%2Fhttps%3A%252F%252Fjournals.plos.org%252Fmentalhealth%252Fs%252Fdata-availability%2F1%2F010f01924ae67f7e-01a4755c-df8e-4cc1-bc38-b44f62b03448-000000%2FeMFcHpq5CuaXIEp9cDh_gj24vRE%3D179&data=05%7C02%7Cpatricia.dekeseredy%40hsc.wvu.edu%7C035b795db0ae4b650fdc08dce283c400%7Ca2d1f95f851044248ae15c596bdbd578%7C0%7C0%7C638634302767010853%7CUnknown%7CTWFpbGZsb3d8eyJWIjoiMC4wLjAwMDAiLCJQIjoiV2luMzIiLCJBTiI6Ik1haWwiLCJXVCI6Mn0%3D%7C0%7C%7C%7C&sdata=G2DZFdDITfv7qZBoDba3KnCgTcyQmfjWI2seHeTYCqM%3D&reserved=0) requires authors to make all data underlying the findings described in their manuscript fully available without restriction, with rare exception. The data should be provided as part of the manuscript or its supporting information, or deposited to a public repository. For example, in addition to summary statistics, the data points behind means, medians and variance measures should be available. If there are restrictions on publicly sharing data—e.g. participant privacy or use of data from a third party—those must be specified. See explanation above.

Reviewer #1: Yes

Reviewer #2: Yes

4. Is the manuscript presented in an intelligible fashion and written in standard English?

PLOS Mental Health does not copyedit accepted manuscripts, so the language in submitted articles must be clear, correct, and unambiguous. Any typographical or grammatical errors should be corrected at revision, so please note any specific errors here.

Reviewer #1: Yes

Reviewer #2: Yes

5. Review Comments to the Author

Please use the space provided to explain your answers to the questions above. You may also include additional comments for the author, including concerns about dual publication, research ethics, or publication ethics. (Please upload your review as an attachment if it exceeds 20,000 characters)

Reviewer #1: 1. A description of the qualitative content analaysis is needed. There so many approaches for content analysis. Please review which one was done. Identify the author of the used qualitative content analysis. We used a conventional content analysis as described by Hsieh & Shannon. We added more descriptions of this process and references. We outlined the step-by-step process and steps to ensure rigor in our analysis as described.
2. Your conclusion need triangulation. We clarified the use of 3 investigators to triangulate our results and strengthen the methodological rigor through the audit trail and a multidisciplinary team (Family Med MD, RN, and Surgeon) at the end of the methods section.

Reviewer #2: Review of "This is an Absolute Witch-Hunt for Nursing, and Nobody Feels Safe": A Qualitative Study of Nurses, Mental Health, and the Criminal Prosecution of a Nurse Error

Introduction
The manuscript titled "This is an Absolute Witch-Hunt for Nursing, and Nobody Feels Safe" provides a poignant and necessary examination of the intricate relationship between nursing practice, mental health, and the legal consequences of errors in a clinical setting. As healthcare becomes increasingly complex and scrutinized, understanding the implications for those on the front lines is vital. This review will delve into the manuscript's methodology, findings, and implications, while also highlighting its strengths and areas for further exploration.

Methodology
The authors adopt a qualitative research design, utilizing in-depth interviews with nurses who have experienced or witnessed the repercussions of legal actions stemming from clinical errors. This approach is particularly effective in capturing the nuanced emotional experiences of the participants, as quantitative data alone would likely fail to convey the depth of anxiety and fear prevalent in the nursing profession. The use of thematic analysis allows for a rich exploration of recurring themes and patterns, which is essential for understanding the collective psyche of nurses facing potential prosecution.

Key Themes
1. Fear and Anxiety

A central theme that emerges from the interviews is the pervasive sense of fear among nurses regarding criminal prosecution. Participants articulate a feeling of being constantly watched and evaluated, which creates an environment where making mistakes can lead to severe consequences, including legal action. This atmosphere of fear is detrimental not only to nurses' mental health but also to patient care. When professionals operate under such pressure, their focus may shift from providing quality care to merely avoiding errors, potentially compromising the very essence of nursing.

2. The Impact on Mental Health

The manuscript effectively highlights the mental health challenges faced by nurses as a result of this fear. Many participants describe feelings of anxiety, depression, and burnout, which are exacerbated by the threat of criminal prosecution. The narrative surrounding mental health in nursing is often overlooked, yet this study brings to light the urgent need for systemic support. The emotional toll of being subject to legal scrutiny can lead to long-term psychological issues, making it imperative for healthcare systems to prioritize mental health resources for their staff.

3. The Stigma of Error

Another significant finding is the stigma associated with making mistakes in nursing. Participants express a strong sense of shame and isolation following an error, often feeling that their professional identity is irrevocably tarnished. This stigma not only affects the individual nurse but also influences the workplace culture, fostering an environment where open communication about errors is stifled. Such a culture can prevent the learning and growth that are essential for improving patient safety and enhancing nursing practices.

4. Systemic Issues and Accountability

The study also touches on the systemic issues within the healthcare system that contribute to the criminalization of nursing errors. Participants discuss how factors such as understaffing, high patient loads, and lack of support exacerbate the likelihood of mistakes. The manuscript argues that addressing these systemic challenges is crucial for reducing errors and the subsequent legal consequences. By shifting the focus from individual blame to systemic accountability, the healthcare system can create a more supportive environment for nurses.

Strengths of the Manuscript

One of the manuscript's strengths is its ability to highlight the psychological toll that such prosecutions can have on nurses, including impacts on mental health and job satisfaction. The stories shared by participants reflect a deep sense of disillusionment and fear, raising important questions about how systemic issues within the healthcare system contribute to individual errors and, subsequently, to punitive measures.

The manuscript excels in its rich, qualitative data, providing a voice to nurses whose experiences are often marginalized in discussions about healthcare accountability. The authors’ ability to weave personal narratives with broader themes enhances the emotional resonance of the study, making it a compelling read for both academics and practitioners.

Additionally, the study's relevance is underscored by the current climate in healthcare, where legal actions against professionals are becoming more common. By addressing this timely issue, the authors contribute significantly to the ongoing discourse surrounding nursing practice, mental health, and legal accountability.

Areas for Further Exploration
While the manuscript provides a comprehensive analysis of the issues at hand, it could benefit from exploring potential solutions in greater detail. Suggestions for interventions, such as the implementation of supportive mental health programs and resources, legal protections for nurses, and training on error management, would enhance the manuscript’s practical implications. Moreover, a discussion on how to foster a culture of learning and transparency within healthcare organizations could provide valuable insights for stakeholders. These are all excellent suggestions. We added a paragraph about just culture that encompasses most these concepts. Implementing just culture is somewhat limited when the error is of interest to the general public. The honest and open reporting of the error (along with some expectation of confidentiality) is lost when the error becomes a “story” for the media. This loss of privacy, along with a public trial, is what drives reporting anxiety and was the crux of this study.

It’s also important for the manuscript to explicitly discuss the limitations of their study, including the potential for selection bias in participant recruitment and the limited generalizability of qualitative findings. By acknowledging these limitations, the credibility of the manuscript would be enhanced. The manuscript's conclusion would be enhanced by offering recommendations for future research that can build upon the findings of this study. By pinpointing specific areas that require further investigation, a valuable roadmap can be provided for future studies. We expanded the limitations to include more detail on selection bias and generalizability. We also included a sentence on future research inspired by your comments above. This was included at the end of the new paragraph on just culture.

The study could also delve into the perspectives of other healthcare professionals and administrators. Understanding how these groups view the issues of error and prosecution would provide a more holistic view of the healthcare environment and inform strategies for improvement. We agree this would be a valuable aspect of the study. Given the impact of media coverage and online chat, we chose to focus on practicing nurses for this study. However, including administrators, managers, and other healthcare professionals could provide more robust insight for future studies.

Conclusion
"This is an Absolute Witch-Hunt for Nursing, and Nobody Feels Safe" is a significant contribution to the literature on nursing, mental health, and legal accountability. By illuminating the fears and challenges faced by nurses, the manuscript serves as a crucial call to action for healthcare leaders and policymakers. Addressing the systemic issues that contribute to the criminalization of nursing errors is essential for creating a safer and more supportive environment for healthcare professionals.

In sum, this study not only sheds light on the pressing mental health concerns within the nursing profession but also emphasizes the need for systemic change to protect and support those who care for our communities. The insights gleaned from this research are invaluable, and further exploration of potential solutions will be crucial for fostering a healthier and more resilient nursing workforce. Thank you!

6. PLOS authors have the option to publish the peer review history of their article ([what does this mean?](https://nam04.safelinks.protection.outlook.com/?url=https%3A%2F%2Fc05y1x9s.r.us-east-2.awstrack.me%2FL0%2Fhttps%3A%252F%252Fjournals.plos.org%252Fmentalthealth%252Fs%252Feditorial-and-peer-review-process%2523loc-peer-review-history%2F1%2F010f01924ae67f7e-01a4755c-df8e-4cc1-bc38-b44f62b03448-000000%2F_jYYQ14fEY8Ek5y8zt896uYy8A4%3D179&data=05%7C02%7Cpatricia.dekeseredy%40hsc.wvu.edu%7C035b795db0ae4b650fdc08dce283c400%7Ca2d1f95f851044248ae15c596bdbd578%7C0%7C0%7C638634302767031149%7CUnknown%7CTWFpbGZsb3d8eyJWIjoiMC4wLjAwMDAiLCJQIjoiV2luMzIiLCJBTiI6Ik1haWwiLCJXVCI6Mn0%3D%7C0%7C%7C%7C&sdata=54hX1VgV%2FgpCGGkvHuhSVWEw6WJ8j26lkGuJpRdkMCY%3D&reserved=0)). If published, this will include your full peer review and any attached files.


**Do you want your identity to be public for this peer review?** If you choose “no”, your identity will remain anonymous but your review may still be made public.


For information about this choice, including consent withdrawal, please see our [Privacy Policy](https://nam04.safelinks.protection.outlook.com/?url=https%3A%2F%2Fc05y1x9s.r.us-east-2.awstrack.me%2FL0%2Fhttps%3A%252F%252Fwww.plos.org%252Fprivacy-policy%2F1%2F010f01924ae67f7e-01a4755c-df8e-4cc1-bc38-b44f62b03448-000000%2FARUHMDP4K2knc9zfMdDetXxpZwU%3D179&data=05%7C02%7Cpatricia.dekeseredy%40hsc.wvu.edu%7C035b795db0ae4b650fdc08dce283c400%7Ca2d1f95f851044248ae15c596bdbd578%7C0%7C0%7C638634302767048116%7CUnknown%7CTWFpbGZsb3d8eyJWIjoiMC4wLjAwMDAiLCJQIjoiV2luMzIiLCJBTiI6Ik1haWwiLCJXVCI6Mn0%3D%7C0%7C%7C%7C&sdata=Ce%2Fnvg6JF9BeuiW1yukt67sB2NWKY76sP5S1n9OR9fI%3D&reserved=0).

Reviewer #1: **Yes:** Prof. Paul Fericelli

Reviewer #2: **Yes:** Dr. Roslyn D. Burton-Robertson, PsyD, PhD, ThD, CNMP, CCTS, CMHP, CP
